# Thiophene End-Functionalized Oligo-(D,L-Lactide) as a New Electroactive Macromonomer for the “Hairy-Rod” Type Conjugated Polymers Synthesis †

**DOI:** 10.3390/polym15051094

**Published:** 2023-02-22

**Authors:** Anca-Dana Bendrea, Luminita Cianga, Demet Göen Colak, Doina Constantinescu, Ioan Cianga

**Affiliations:** 1 “PetruPoni” Institute of Macromolecular Chemistry, Centre of Advanced Research in Bionanoconjugates and Biopolymers, 41A, Grigore–GhicaVoda Alley, 700487 Iasi, Romania; 2 Department of Chemistry, Faculty of Science and Letters, Istanbul Technical University, 34469 Istanbul, Turkey; 3 MONOFIL SRL, Str. Gheorghe Caranfil nr. 5F, 617410 Savinesti, Romania

**Keywords:** electroactive macromonomers, grafted conjugated polymers, polylactide, oligothiophenes, photopolymerization, iodonium salt

## Abstract

The development of the modern society imposes a fast-growing demand for new advanced functional polymer materials. To this aim, one of the most plausible current methodologies is the end-group functionalization of existing conventional polymers. If the end functional group is able to polymerize, this method enables the synthesis of a molecularly complex, grafted architecture that opens the access to a wider range of material properties, as well as tailoring the special functions required for certain applications. In this context, the present paper reports on α-thienyl-ω-hydroxyl-end-groups functionalized oligo-(D,L-lactide) (**Th-PDLLA**), which was designed to combine the polymerizability and photophysical properties of thiophene with the biocompatibility and biodegradability of poly-(D,L-lactide). **Th-PDLLA** was synthesized using the path of “functional initiator” in the ring-opening polymerization (ROP) of (D,L)-lactide, assisted by stannous 2-ethyl hexanoate (Sn(oct)_2_). The results of NMR and FT-IR spectroscopic methods confirmed the **Th-PDLLA**’s expected structure, while the oligomeric nature of **Th-PDLLA**, as resulting from the calculations based on ^1^H-NMR data, is supported by the findings from gel permeation chromatography (GPC) and by the results of the thermal analyses. The behavior of **Th-PDLLA** in different organic solvents, evaluated by UV–vis and fluorescence spectroscopy, but also by dynamic light scattering (DLS), suggested the presence of colloidal supramolecular structures, underlining the nature of the macromonomer **Th-PDLLA** as an “shape amphiphile”. To test its functionality, the ability of **Th-PDLLA** to work as a building block for the synthesis of molecular composites was demonstrated by photoinduced oxidative homopolymerization in the presence of diphenyliodonium salt (DPI). The occurrence of a polymerization process, with the formation of a thiophene-conjugated oligomeric main chain grafted with oligomeric PDLLA, was proven, in addition to the visual changes, by the results of GPC, ^1^H-NMR, FT-IR, UV–vis and fluorescence measurements.

## 1. Introduction

Polymer materials play an irreplaceable role in the daily life and their appearance definitely shaped the technological evolution. As a future world without polymers is unthinkable, the development and discovery of the so-called “advanced functional polymer materials” with new properties and applications will be one of the most important topics for the next period [1]. For example, there is a stringent need for advanced polymer materials in the area of future electronic devices. There are no doubts that the “electronic revolution” of the 20th century impacted almost every aspect of human life and the social benefits of modern electronics cannot be underestimated [2]. Today, almost every aspect of the modern human endeavor is influenced on some level by electronic devices, and the rapid evolution of electronic information technology shifted the research interest toward the flexible electronic devices [3,4]. Having skin-inspired properties (particularly light weight, biocompatibility, stretchability, conformability, biodegradability), this new generation of electronic devices is offering unlimited possibilities for seamless integration with the natural world [5], as the electronic components become fully portable, wearable and even implantable. Thus, the enhancing of our environmental perception and of the interactions is expected, while new markets in personalized patient care, environmental monitoring, consumer products and networked information could be opened [6].

Polymers have the potential to drive research progress in this emerging paradigm of electronics, because most of the specific requested materials’ properties can be accessible through the so-called “plastic/organic electronics” [7,8]; they are partially or totally of “all-polymer” type [9,10,11,12], and their active layers are based on π-conjugated, electronoconducting polymers (CPs) (“organic semiconductors”) [13,14].

Supported by decades of fundamental research, CPs are the main components of a natural “bridge” between electronics and soft matter [15]. Polymer design and various synthesis/processing technologies being the major driving force and foundation for future plastic electronics [16], both can mediate the improvement in CP performances and the development of a variety of new integrated functions [17]. However, conferring all the skin-like properties to CPs-containing materials is a non-trivial task. And, this in spite that a set of intrinsic attributes of CPs (like softness, carbon-based molecular structures, shape persistence, self-assembling propensity) gives them a biomimetic character through structural and functional similarities to biological systems [18,19], to which can also be added their unique capability of coupling between electronic and ionic charges transport. Such a combination facilitates efficient interfacing with the inherently ionic living systems [20]. Moreover, it is an increased demand not only for an enhanced biocompatibility of materials but also for transience [21,22], as flexible electronic devices extend their applications area toward organic bioelectronics (devices which exploit the functional versatility of CPs to transduce biological events into electronic signals) [23]. The integration of suitable biomaterials as structural components in organic electronic devices has the potential to provide a viable solution [24,25], enabling a paradigm shift not only for temporary biomedical implants, but also for “green” consumer gadgets or smart packaging [2,26]; as such, the use of common biodegradable/bioresorbable polymers, particularly synthetic ones, is increasing in the field of transient electronics [25].

In this context, based on the CPs’ ease of chemical functionalization [23] and using clever chemistries coupled with the advances in processing, an effective integration of biomaterials with CPs proved to be feasible by covalently connecting them in different topological formats [27,28,29,30,31,32,33,34,35]; this giving rise to the CPs-based electroactive biomaterials, as a new generation of “smart” biomaterials [36]. End-group functionalization of well-known synthetic polymers-based biomedical materials (polyethyleneglycol–PEG, poly-ε-caprolactone–PCL, polylactide–PLA, etc.) was one from the adopted synthesis approaches [27,28,29,30,31,32]. There are several reasons why this strategy was a rational and a successful starting step. Therefore, in addition to being a large-scale way to develop new polymer-based functional materials with well-defined end-functionality, the controlled polymerization methods applied for their synthesis [37,38] provide polymers/oligomers with a precisely controlled molecular weight. On the other hand, these end groups that influence the already existing properties of the polymers to which they are attached [39,40,41] and/or induce new ones at the molecular and/or supramolecular level [42,43,44,45,46], more importantly, have the ability to transform these polymers/oligomers into macromonomers [47,48], if they are polymerizable. The introduction of the concept of “macromonomers” in polymer chemistry allowed for the easy access to grafted (co)polymers. This polymeric architecture, which initially was thought of as a viable alternative to surface property manipulation techniques [49], later evolved to “cylindrical polymer brushes”, a category working as a versatile toolbox for next generation nanomaterials [50].

Placing an electrochemically polymerizable moiety as a final functional group to different types of flexible polymer chains was a very innovative idea, later extended also to other types of CPs’ polymerization methods [51,52,53,54,55], significantly impacting the development of CPs.

This is because the polymerization of electroactive macromonomers leads to grafted CPs (g-CPs) (or “hairy-rod” CPs), which are essentially characterized by the chemical and stiffness mismatch between the conjugated, rod-like main chain and the flexible side chains. Such contrast is significant due to the complex effect on the hierarchical self-assembly pathway in solution during processing. From this viewpoint, the g-CPs’ architecture is suitable for different types of applications, allowing the control of the processing–structure–properties relationship from the molecular design stage, when the self-assembly can be programmed and structurally optimized [56]. Defined as “molecular composites” in order to underline that their components are dispersed at the molecular level, g-CPs’ performances can be adjusted and improved not only by conjugated main chains type manipulation, but also by changing the well-defined side-chain grafting density, lengths or nature [27,33,57,58,59,60,61,62]. Moreover, g-CPs can be processed from solution by drop-casting [29] and ink-jet printing [63], electrospinning [64,65] and by 3D printing [66], in various form factors such as nanoparticles [59,67], thin films [29,68,69], nanofibers [64,65] or 3D constructions [30,66]. Taken together, all of these features allowed g-CPs to become a promising family of materials for diverse biomedical applications. Among all CPs classes, polythiophenes (PTh)s proved to be some of the most suitable [70,71]. Specifically, grafted (PTh)s (g-PTh)s or g-PEDOTs (the PTh derivative) have been synthesized and investigated for different bioapplications. For example, such materials can work as excellent biocompatible and electroactive cellular scaffolds [30,61,66,69,72], or as active surfaces for selective proteins adsorption [27]. Their use for cell imaging [59] or for sensing of various bioanalytes [28,29,31,73,74] was demonstrated, as well.

Based on the importance of electroactive biomaterials derived from PTh and on the advantage that the structural details of the macromonomers are reflected in the properties of the materials built on their basis, in this work our interest was directed to combining the polymerizability of the thiophene ring with the biocompatibility and biodegradability of polylactide in a new thiophene-containing macromonomer denoted **Th-PDLLA**. Among synthetic polymers, PLA is a promising bio-resourced one, a valuable alternative to petroleum-based plastics within the green chemistry context. Widely used in day-to-day and technological applications, PLA and its copolymers are ideal biomedical materials due to good mechanical integrity, biocompatibility and its propensity for hydrolytic degradation in physiological media [41,48]. Based on its structural peculiarities, **Th-PDLLA** is expected to function as a precursor for the construction of g-CPs endowed with electrochemical and/or electro-bio-activity, alternatively showing photo- and/or electroluminescence, simultaneously capable of adequate functioning in the aqueous phase under physiological conditions, being also potentially disintegrable.

In this current report, besides details referring to synthesis and to basic characterization of **Th-PDLLA**, its capability for g-CPs synthesis was also demonstrated by metal-free photoinduced oxidative homopolymerization. The obtaining of a grafted thiophene oligomer was proven by GPC, ^1^H-NMR, FT-IR, UV–vis and fluorescence measurements.

## 2. Materials and Methods

### 2.1. Materials

The 3-thiophene-methanol (Th-Me), 3,6-dimethyl-1,4-dioxane-2,5-dione (or *rac*-lactide, the 50:50 racemic mixture of D- and L-Lactide, accordingly with provider description), tin(II)-2-ethylhexanoate (Sn(Oct)_2_) and diphenyliodonium hexafluorophosphate (DPI) (>98%) (all from Merck-Sigma–Aldrich, Darmstadt, Germany) were used as received. All the used solvents were purified and dried by usual methods.

### 2.2. Synthesis

#### 2.2.1. Synthesis of PLA-Based Macromonomer (**Th-PDLLA**) by Ring-Opening-Polymerization (ROP)

The 3,6-dimethyl-1,4-dioxane-2,5-dione (D,L-lactide), (3 g, 0.0208 mol), **Th-Me** (0.123 mL, 0.0013 mol) and Sn(Oct)_2_ (0.0084 g, 0.208 × 10^−4^ mol) were added under nitrogen into a previously flamed and nitrogen-purged two-neck round-bottom flask equipped with a dropping funnel and magnetic stirrer. The flask was heated while stirring to 120 °C. After 30 h, the resulting mixture was diluted with dichloromethane (CH_2_Cl_2_) and poured into a tenfold excess of cold methanol. The obtained macromonomer **Th-PDLLA** was collected after filtration and dried at room temperature in vacuum for 3 days. Further purification was performed by passing its solution in CH_2_Cl_2_ through a silica-gel filled column and then reprecipitated in cold methanol.

#### 2.2.2. Synthesis of **OTh-PDDLA** by Photochemical Oxidative Polymerization of **Th-PDLLA** Macromonomer

The photopolymerization of the **Th-PDLLA** macromonomer (as a white powder in Figure S5A) was carried out by adapting the already reported procedures as those used for bare thiophene and for 3-hexylthiophene [75,76]. Thus, prior to irradiation, a solution of 0.427 mg of **Th-PDLLA** (0.17 mmol calculated based on the value of M_n_ as resulted from ^1^H-NMR) in 1.5 mL of CH_2_Cl_2_ as the solvent and an amount of 0.0244 g (0.057 mmol) of DPI as onium salt were placed in a quartz cell. The reaction proceeded under nitrogen atmosphere by irradiation with a lamp emitting light nominally at 300 nm (Hamamatsu Lightningcure Type LC8, Model L9588). During the first 12 h of reaction the solution kept its liquid appearance, with the only change of the color toward light brown. However, at the end of the reaction interval (24 h) a solid mass appeared in the cell, having a darker color. This solid mass was transferred into a flask containing 15 mL of CH_2_Cl_2_ and kept under stirring for several hours. Then, the solid fraction (denoted as **OTh-PDLLA** in Scheme S1) with a glassy brownish aspect (see Figure S5B) was obtained after the separation by filtration and separate washing with methanol. It was considered as the main product of the photoinduced polymerization. Further, the remaining solution in CH_2_Cl_2_ was worked up, as is schematically explained in Scheme S1. Another two solid fractions, (denoted as **F1** and **F2** in Scheme S1), containing low oligomers (as was identified by ^1^H-NMR spectroscopy, Figure S6), were separated as well.

### 2.3. Measurements

The NMR spectra were recorded at room temperature on a Bruker Avance DRX-400 spectrometer (400 MHz) (Bruker Biospin, Ettlingen, Germany) in CDCl_3_ and chemical shifts are reported in ppm and referenced to TMS as internal standard. The apparent molecular weights (M_n_) and molecular weight distribution (reflected in index of polydispersity-IPD) of the synthesized compounds (**Th-PDLLA** and **OTh-PDLLA**) were determined by gel permeation chromatography (GPC). A Waters 515 instrument, at a flow rate of THF eluent of 0.3 mL/min and monodisperse polystyrene standards for the calibration plot, was used for **Th-PDLLA**. The measurements in chloroform (Chl) for both **Th-PDLLA** and **OTh-PDLLA** were performed by using a WGE SEC-3010 multidetection system, consisting of a pump, two PL gel columns (PL gel 5 micro Mixed C Agilent and PL gel 5 microMixed D Agilent), and a dual detector refractometer/viscometer (RI/VI) WGE SEC-3010 at a flow rate of 1.0 mL/min. The RI/VI detector was calibrated with PS standards (580–467,000 DA) having narrow molecular weight distribution. The system was also equipped with a UV detector WGE SEC-3010 and Bi-MwA Brookhaven multi-angle SLS detector. Data were analyzed using PARSEC Chromatography software. The FTIR spectra were recorded on a Bruker Vertex 70 FTIR spectrometer (Bruker, Ettlingen, Germany) in transmission mode, by using KBr pellets. Measurements of UV–vis and of fluorescence for the reported compounds, in three organic solvents, (Chl, tetrahydrofuran–THF and acetonitrile-ACN), were carried out by using a Specord 200 Analytik Jena spectrophotometer (Analytik Jena AG, Jena, Germany) and Perkin Elmer LS 55 apparatus (PerkinElmer, Inc., Waltham, MA, USA), respectively. The concentration used was kept constant as 1 mg/mL, for **Th-PDLLA** being equivalent with 0.4 × 10^−3^ M, based on the value of M_n-NMR_, (as calculated from the data of ^1^H-NMR registration).

DSC experiments were conducted on a Perkin Elmer 4000 DSC apparatus (PerkinElmer Inc., Shelton, USA) calibrated with indium. Around 10 mg of each sample was weighed in pressed and punched aluminum crucibles. Nitrogen was used as inert atmosphere at a flow rate of 50 mL/min. Heating and cooling rates of 10 °C/min were applied. The melting temperature was taken as the maximum of the endothermal melting point, while the glass transition temperature (*T*_g_) was taken as the mid-point on the curve showing the heat capacity change. TGA measurements were performed on Perkin Elmer Diamond TGA/DTA equipment (PerkinElmer Inc., Shelton, USA). Around 10 mg of each sample was weighed in aluminium crucibles with no lids. A heating rate of 10 °C/min was applied. Nitrogen was used as inert atmosphere at a flow rate of 50 mL/min.

Particle characterization was carried out by dynamic light scattering (DLS) using a Delsa Nano C Submicron Particle Size Analyzer (Beckman Coulter, Inc., Fullerton, CA, USA) equipped with dual 30 mW laser diodes emitting at 658 nm. The intensity weighted mean hydrodynamic size (Z average) and the polydispersity factor were obtained from analysis using the autocorrelation function. The **Th-PDLLA** sample in Chl was used as prepared, without filtration, at a concentration of c = 1 mg/mL placed into a quartz cell. The reported values represent the average of three measurements performed at 25 °C with an equilibration time of 5 min before starting each measurement.

## 3. Results and Discussion

### 3.1. Synthesis and Structural Characterization of **Th-PDLLA**

While the specialized literature abounds in examples of composites/blends or coatings based on PLA and conjugated polymers generally used for electronic devices (like supercapacitors, solar cells, flexible 3D printed electrodes or actuators [77,78,79,80]), or alternatively, for tissue engineering, platforms for therapeutics delivery or as multifunctional biomedical surfaces [81,82,83], surprisingly, there are only a few articles referring to conjugated polymers grafted with PLA side chains [32,66,69,84,85,86,87,88]; these date back no more than about a decade. Most of them are reporting on the design and on the resulted polymer’s basic properties, and only recently the potential of PEDOT-*g*-PLA for applications in tissue engineering was assessed [66,69].

**Scheme 1:**
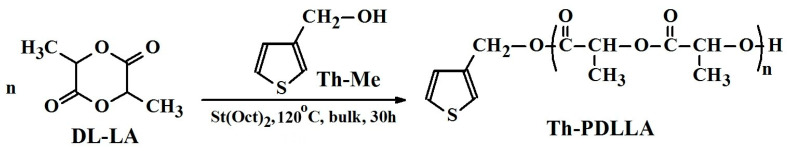
Synthesis pathway of **Th-PDLLA**.

In addition, the “macromonomer technique” was dominantly used for these CPs-*g*-PLA syntheses, via the “functional initiator” path [32,66,84,85,86,87]. Based on this method, in our attempt the commercially available **Th-Me** in conjunction with FDA-approved Sn(Oct)_2_ were employed for initiating the ROP reaction of racemic lactide (D,L-lactide), in bulk, as illustrated in Scheme 1.

Our choice for the racemic form of the lactide monomer was guided by the intention to use the **Th-PDLLA**-derived polymers in construction of transient bionic interfaces. Thus, it was taken into account the higher hydrolytic (bio)degradation rate of poly(D,L-lactide) (PDLLA) in comparison with PLLA (derived from L-lactide, with a higher degree of crystallinity) in biological media.

Furthermore, the value of [M]/[I] ratio into the feed was adjusted in favor of lactide oligomers formation, whose biodegradation rate is also known to be higher [69].

The successful synthesis of **Th-PDLLA** with controlled molecular weight, well-defined end-functionalization and narrow molecular-weight distribution (IPD = 1.18) was confirmed from the NMR, FTIR and GPC results.

**Figure 1:**
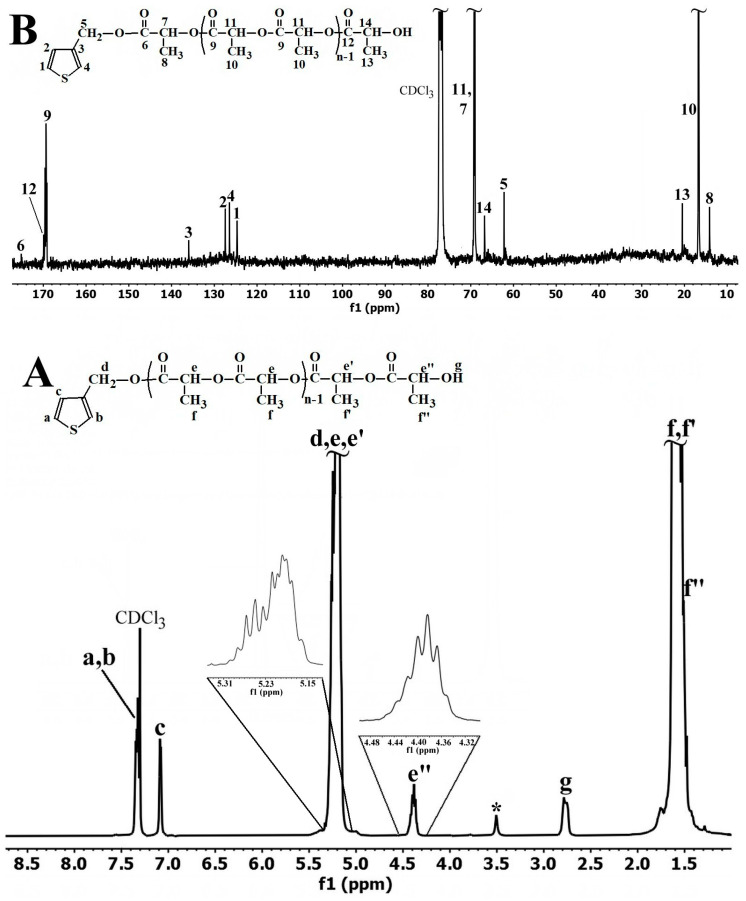
(**A**) ^1^H-NMR and (**B**) ^13^C-NMR spectrum of **Th-PDLLA** registered in CDCl_3_ (with * were denoted the impurities, possible traces of methanol).

In Figure 1A, the ^1^H-NMR spectrum in CDCl_3_ reveals the typical features of an oligomeric PDLLA, showing an undefined multiplet in the range 5.15–5.31 ppm which was attributed to the methine type protons **e** and **e’**, associated with the main chain lactidyl repeating units and that from the chain’s terminal unit placed in their immediate neighborhood. This multiplet is characterizing a most probable atactic stereosequence of D and L units, being in stark contrast to the perfect quadruplet of the methine protons, specific to the stereoregular PLLA [89]. In fact, this signals complicated shape, is also due to the appearance in the same region of the methylene protons **d**, derived from the initiator **Th-Me**, which change their position from δ 4.5 ppm in **Th-Me** [90] to a lower field, at 5 ppm region. This shifting is considered as a piece of evidence which supports the **Th-PDLLA** formation. The irregular quartet present in the range 4.32–4.48 ppm corresponds to the methine proton **e”** in the terminal lactidyl unit, belonging to the –CH group that is directly connected to the ω-terminal hydroxyl function. In the range from 1.53–1.63 ppm are placed the peaks associated with the protons of the methyl groups (**f**, **f’** and **f”**) corresponding to the main oligomer chain (**f** at 1.6 ppm), and also of the terminal lactidyl unit (**f’** and **f“**). In the aromatic region the signals for all types of protons (**a**, **b** and **c**) of the thiophene ring originating from the initiator can be observed. These signals keep roughly the same position as those of **Th-Me**, the independent and clearly visible peak of the **c** proton appearing at about 7.1 ppm. In addition, the peak of the hydroxyl proton **g** at the ω-chain end is distinctively observed in this spectrum at δ = 2.8 ppm. The evidence for the formation of the macromonomer’s expected structure is also supported by the results of the ^13^C-NMR spectrum (Figure 1B). For the carbon atoms of the thiophene ring, four peaks can be distinguished at 124.65 ppm, 126.42 ppm, 127.40 ppm and at 135.96 ppm, with the atoms being assigned in the order **1**, **4**, **2** and **3**, respectively.

If, for the atoms **1**, **4** and **2** a slight down-field shifting is recorded compared to the position of similar atoms in the initiator **Th-Me** (121.71 ppm; 126.28 ppm; 127.40 ppm) [90], for the quaternary carbon atom **3**, placed in the immediate vicinity of the oligolactide chain, a significant up-field shifting from 142.28 ppm to 135.96 ppm was noticed. This shifting can be attributed to the changing of the environment around carbon **3** by the modification of the substitute type at the 3rd position of the thiophene ring; this being one of the strongest evidences supporting **Th-PDLLA** obtainment. Moreover, for the carbon atom **5** of the methylene group directly connected to the thiophene ring, the signal at 62.2 ppm in the spectrum was attributed; this only slightly differs from the position of its counterpart at 60 ppm in **Th-Me** initiator [90].

Representative signals for the oligomeric lactide chain appeared in the methyl, methine and carbonyl zone of the spectrum. Thus, for the carbon atoms **9**, **10** and **11** belonging to the main chain repetitive structural units, the signals at 169.34 ppm, at 16.68 ppm and that at 69.04 ppm were assigned. The peaks attributable to the carbons of methine groups (**7** and **14**), placed toward α- and ω-chain ends, can be noticed at 69.21 ppm and 66.75 ppm, respectively. For carbon atoms **9**, **10**, **11** and **14** the found positions coincide with the already reported ones [89,91]. While peaks characteristic to carbons **8** and **13** of methyl groups flanking the main lactidyl structural units appeared at 14.11 ppm and at 20.52 ppm, for the carbon atoms of carbonyl esters **6** and **12** the signals at 175.12 ppm and at 169.88 ppm were assigned. These values are in agreement with the previously reported ones [89]. In the carbonyl zone, extra undefined signals that can be attributed to the possible presence of different tetrads were also noticed in the range 169.13–169.89 ppm, in a similar manner as previously shown [92].

Not only the expected structure of **Th-PDLLA** macromonomer can be confirmed from the ^1^H-NMR results, but other important data regarding its properties can be obtained as well. For example, when calculating the peak area ratio of the α-terminal **c** proton to the ω-terminal methine **e’** proton, a value of 1 is obtained, attesting to a nearly 100% efficiency of **Th-Me** as an initiator in the ROP of D, L-lactide. Comparing the area of the peaks in the range 5.15–5.31 ppm, corresponding to all protons of type **e** in the main chain plus three more protons (one proton **e’** and two protons **d**), with the area of the peak of proton **c** or, alternatively, with the area of the proton **e”**, allowed us to obtain the value of the degree of polymerization (DP) and subsequently to evaluate the molecular weight (M_n-NMR_) of the obtained oligomeric PLA chain. The resulting values are PD = 16.5 (close to the designed value and corresponding to 33 lactate repeating units) and M_n-NMR_ = 2489.

The GPC elution curves of **Th-PDLLA**, registered in two different solvents (Figure S1), showed monomodal shape. While significantly different results were obtained regarding to M_n_ values in the two solvents (1943 in THF and 2463 in Chl), the values for IPD were practically identical, around 1.2. Moreover, the measured values of M_n-GPC_ obtained in both solvents are lower than the calculated M_n-NMR_, in particular when THF was used as eluent (see Figure S1).

For oligo- and polylactides, especially when the α end-groups are of linear aliphatic nature, the difference between the M_n-GPC_ and M_n-NMR_ values is usual [93]. The difference between the hydrodynamic characteristics of the polystyrene standards used for GPC calibration and those of PLA was cited as the motivation for this discrepancy and for the overestimation of the M_n-GPC_ values [93]. However, in the case of bulky, photosensitive, aromatic terminal groups, in addition to overestimation [86] a tendency for M_n-GPC_ underestimation was also observed [32,43]. It seems that not only the reason mentioned above, but especially in solution self-assembly tendency already noticed for such compounds [41,44,46], which affects the GPC measurement, have to be considered in addition to explain the discrepancy between the two methods used for M_n_ evaluation.

The structural characterization of **Th-PDLLA** was also completed by FTIR measurement. The spectrum is given in Figure S2, and the values of the main appearing bands, as well as their assignment based on already reported results [28,29,53,94,95], are given in Table 1.

As expected, the typical values for both oligolactide chain and thiophene ring were identified. Thus, the band that appeared at 3516 cm^−1^ is associated to the presence of the hydroxyl ω chain-ends in resulting oligolactide, and its appearance with a high intensity confirms once more the oligomeric nature of chain. The strong IR bands at 2998, 2943 and 2880 cm^−1^ are associated with the C-H stretching vibration. At 1755 cm^−1^ a broad asymmetric band appeared assignable to C=O stretching in the ester functionalities, while the signal as a shoulder at 1617 cm^−1^ was attributed to the C=C stretching vibrations of the thiophene ring. The bands at 862, 831, 795 and 699 cm^−1^ were also assigned to the thiophene heterocycle, corresponding to C-S linkages. The CH deformation and asymmetric bands appeared at 1383 cm^−1^ and 1366 cm^−1^. Furthermore, the C-H bending modes results in the band at 1321 cm^−1^ and that at 1272 cm^−1^ which overlaps with the C-O-C ester stretching. Interestingly, two peaks at 955 cm^−1^ and at 916 cm^−1^, which were considered characteristic of the PLA helical backbone vibrations with CH_3_ rocking mode, are discernible in the spectrum as well.

These results are in line with those from the NMR registration, supporting the obtainment of the new macromonomer.

### 3.2. Properties of **Th-PDLLA** in Solution and in Bulk

The physical properties of PLA in general [96], and of end-group functionalized PLA in particular [41], are influenced by many factors like stereoregularity and stereosequences distribution [96,97], the molecular weight [98,99], and obviously by the functional end groups [39,40,43,45,97]. These influences are more pronounced when the lactide oligomers are considered [100,101]. Consequently, the next section is devoted to the investigation of how the placing of the thiophene ring as α end-group is reflected on the properties in solution and in bulk of the resulted ODLLA.

#### 3.2.1 The Behaviour of **Th-PDLLA** in Different Organic Solvents

The new macromonomer is constructed as an amphiphilic homopolymer and also as a “shape amphiphile” (see Scheme 1 and Graphical Abstract), categories that are known to self-assemble in solution into ordered supramolecular morphologies, even if they do not follow the traditional self-assembly rules of copolymers, but mostly due to subtle effects of the terminal hydrophobic groups [44,102]. As such, the photophysical properties of **Th-PDLLA** were considered important in the context of the paper aims and in particular fluorescence measurements.

This technique can give information on the product’s self-assembling behavior, if it exists, being very sensitive to those changes that occur as a result of a change in the solute conformation due to the change in the solvent. Consequently, the absorption and emission properties were followed in three organic solvents: Chl and THF as good ones for PLA but differing between them by polarity, while ACN is a solvent on the borderline of PLA solubility.

**Figure 2:**
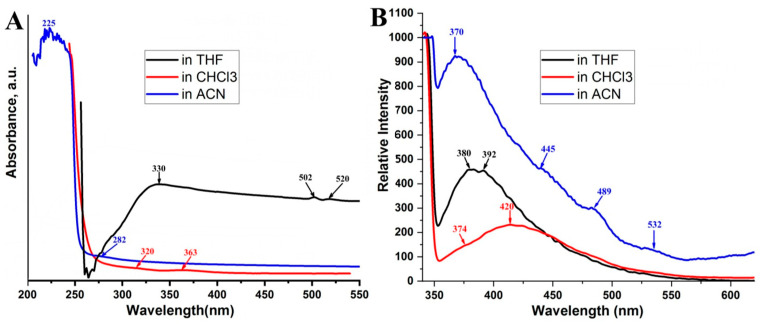
Absorption (**A**) and emission (**B**) spectra of **Th-PDLLA** in organic solvents of different nature (c = 1 mg/mL) (λ_ex_ = 330 nm).

It has been reported that in thin film [103] or in solution [39] PLA has an absorption band in the UV range at 230 nm/250 nm due to the carbonyl from the ester groups. On the other hand, thiophene, in various solvents, shows an absorption maximum around 231 nm [104]. If the traces of UV–vis measurements in Figure 2A are considered, it can be seen that an absorption maximum with a vibronic appearance and a width of about 30 nm, centered at 225 nm, appeared in ACN. Because of the UV cutoff this absorption was not discernible in the other two solvents. It can be concluded that on this spectrum interval there is no mutual impact on the absorption property of any of the structural components of **Th-PDLLA**, and that the appeared absorption maximum could be mostly due to the combined effect between the absorption of the thiophenic end group and also of the oligolactide chain. Additionally, in the range 270–550 nm in Figure 2A, in all the investigated solvents several shallow bands appeared, of low intensity, with a more defined identity in Chl (320 nm, 363 nm) and THF (330 nm, 502 nm, 520 nm). In fact, the presence in itself and also the shape of these absorptions could be indicative for the formation of the self-assembled structures of micelle type, as previously reported [44,46].

Moreover, the different and less usual shape of the UV–vis trace in THF in Figure 2A, in particular in the range 300–600 nm, could be due to the phenomenon of light scattering. The eventually formed colloidal particles by the self-assembling in this solvent could be the source and the motivation for the light scattering, which interferes and hinders the accurate UV–vis investigation (see Supplementary Materials for details).

To prove the self-assembling and block-like character of **Th-PDLLA**, the DLS registration in Chl at the same concentration as that used for UV–vis and fluorescence measurements was performed, and the result is given in Figure S3. The attention was focused on Chl because of its highest similitude of the solubility parameters with those of PDLLA, but more specifically because the value of χ parameter, which is the lowest of all of the used solvents (see Table S1), confirms the best compatibility with the lactide oligomer. From the result of the registration in Figure S3, it indicates that **Th-PDLLA** forms self-assembled structures in Chl. Having multimodal shape, the DLS result is advocating for a mixed population of particles with size of apparent hydrodynamic diameter (D_h_) between several tens and thousands of μm, the highest size being suggestive for the formation of particle clusters. As the values of χ parameter are almost similar, the reason of the self-assembly could be mostly due to shape dissimilarity between the constitutive parts of the macromonomer.

In Figure 2B are given the curves obtained from the fluorescence measurements of the same solutions which were excited with a wavelength having a median value (λ_ex_ = 330 nm) of those that appeared in the traces of all the solvents with low intensity. Surprisingly, all the samples fluoresced and the obtained traces are of non-symmetrical shape and broad, covering a range of approximately 200 nm and showing emission maxima placed in the blue region of the visible light (370 nm–535 nm). The changes in the photoluminescence behavior in the three solvents seemingly correlate with the change in the solvents’ dielectric constant. Thus, a hypsochromic shifting associated with an increase in the emission intensity can be noticed when the dielectric constant (ɛ) of the solvents increases (see Table S1 for the ɛ exact values). A difference in shape, intensity and position of emission peaks between Chl and THF compared to ACN can be seen in Figure 2B. This suggests distinctly different self-assembling behavior in the marginal ACN than in both other cases. Not only the position and the values of the intensities of the appeared emission peaks maxima are different in ACN, but also their number (Figure 2B). The explanation behind all of these observed phenomena goes beyond the statement that PLA does not fluoresce [105], in the commonly used “classical” sense, and also that Th does not fluoresce but exhibits phosphorescence [104]. It can be found and is related to the increasingly interesting topic of the atypical light-emitting materials based on non-conjugated and non-aromatic structures, which show clustroluminescence, a phenomenon taking place in aggregated/clustered state and/or under physico-chemical confinement [106]. In this context, it was reported that carbonyl-based aliphatic polymers (with polyesters as representative) show visible luminescence and are the ideal candidates for fluorescence property manipulation [107]. Moreover, in this very recent study, Ref. [107], it was experimentally found that a CH_2_Cl_2_ solution of 0.1M of PLA (M_n_ unmentioned) fluoresced at 440 nm and shows the feature of the clusteroluminescence property of a λ_excitation_-dependent fluorescence. The phenomenon originated from (n, π^*^) transition of carbonyl of ester groups and due to “through-space” interactions and, essentially depend on chain conformational flexibility. Since the concentration used for the results shown in Figure 2 (0.4 × 10^−3^ M) was incomparably lower than 0.1 M used in [107], our overall results, obtained from fluorescence measurements, lead to the conclusion that the particular interactions of ODLLA (as a major component of the **Th-PDLLA** structure), with each of the used solvents, determines its resulting conformation (more collapsed or stiffened), which is an important factor influencing this property. In addition, the specific planar geometry of Th and the particular interaction between the rings, in addition to their relative orientation, which is determined by the aromatic nature of Th and its steric characteristics, may also contribute to the observed behavior. Most likely, the photoluminescence of **Th-PDLLA** is due to the self-assembly phenomenon, and it depends on the conformation of the ODLLA chains and, very importantly, on their mutual placement in the formed supramolecular confinement—placement which is practically driven by the size and the shape of the formed self-assembled structures. However, in order to shed light on this important aspect, detailed investigations are necessary which constitute a study in itself and which exceeds the scope of this paper. All the above results showed that the presence of the thiophene ring as α end group impacted the behavior of the ODLLA from photophysical properties viewpoint.

#### 3.2.2. Thermal Properties of **Th-PDLLA**

**Figure 3:**
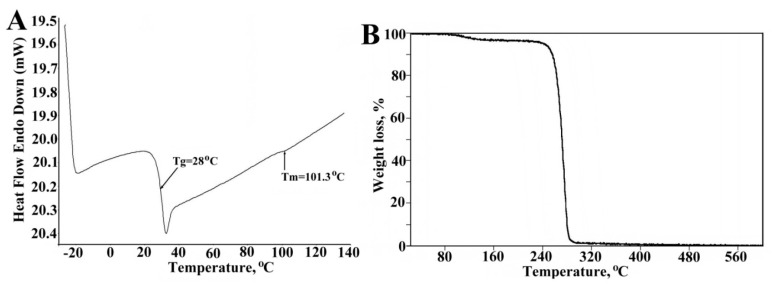
DSC trace of treated (**A**) and TGA trace of untreated (**B**) **Th-PDLLA** macromonomer.

In order to get more information about how the thiophene end group influences the thermal properties of the formed oligomeric lactide, **Th-PDLLA** was analyzed in bulk by DSC and TGA, and the results are given in Figure 3 and Figure S4. This is all the more so since, as reported, the influence of the final group is very significant [93,108], especially in the case of oligolactides α-ended with aromatic and bulky groups [97,101]. The temperature range for DSC heating was established between −30 °C or −20 °C and maximum 220 °C (Figure 3A and Figure S4), based on the results of the thermal stability of **Th-PDDLA** obtained by TGA registration (Figure 3B).

The DSC investigation was performed on two samples; one of them by using the product as resulting after the purification and keeping in desiccator under vacuum, and another one on the sample taken from desiccator after a while and to which a 48 h treatment into the vacuum oven, at room temperature, was applied (denoted as “treated sample”). The T_g_ values obtained for both types of **Th-PDLLA** samples (26.5 °C for the untreated one and 28 °C for the treated one) are in agreement with the values reported for other PLA end-functionalized compounds with molecular weight in the range of oligomers [97,98,99]. This comparison reconfirms the conclusion that the final group has a minimal (if not absent) influence on the T_g_ value, this being dependent only on the molecular mass value [100].

On the other hand, PDLLA is generally an amorphous polymer, showing only T_g_ due to the heterotacticity. By contrast, pure enantiomers PLLA and PDLA are semicrystalline showing both T_g_ and melting temperature (T_m_). PDLLA can exhibit some crystallinity when synthesized by stereocontrolled ROP through the action of the catalyst/initiator complex [96]. However, in a few cases, despite the use of (D,L)-lactide as monomer and Sn(Oct)_2_ as catalyst, the resulting compounds were reported to exhibit, beside T_g_, also T_m_, but no explanation was provided for this behavior [105,109,110]. Surprisingly, in the DSC experiment of the untreated **Th-PDLLA** sample (Figure S4), the shape of the endotherm of the first heating cycle, starting around 100 °C and ending at around 194 °C, suggests an overlapping phenomenon that could be attributed to a melting and/or evaporation of traces of water, as moisture sorption is a feature favored by the low molecular weight and amorphous state of PLA [111]. In fact, the TGA analysis showed that the weight loss, which developed in the same temperature range (Figure 3B), has a calculated value of only 3.92% which advocates for a possible release of a small amount of adsorbed water.

Our claim could be also supported by the result obtained in DSC measurement of the treated **Th-PDLLA** sample, in the trace of which only a shallow endotherm (a shape often appearing in end-group functionalized PLA [108]), centered at approximately 101 °C was noticed (Figure 3A). Moreover, for untreated **Th-PDLLA**, after the first cooling cycle in which only the vitrification phenomenon occurred, (evidenced by the signal at 32 °C), in the second heating cycle, in addition to T_g_ (which appeared at a lower value of 25.3 °C), two consecutive signals centered at 67 °C and 72 °C, respectively, were discernible, which could suggest a combination of a cold crystallization followed by a melting phenomenon. Such a behavior mimicks that of end-functionalized PLLA [101]. It is, like **Th-PDLLA**, resulting from synthesis with an isotactic-enriched microstructure, which could be the result of the using **Th-Me** as initiator. It could be possible that the particular steric interactions and/or electronic effects, (arising in the catalytic complex comprised of initiator, metal atom, coordinated lactide molecule and PLA growing chain), to significantly influence the stereoselectivity of ROP polymerization. At this point the motivation for the found thermal behavior of the new macromonomer can only be speculative, but a homonuclear decoupling NMR study for microstructure analysis and circular dichroism measurements is on the way which, hopefully, will allow for a future explanation based on experimental findings.

Regarding the thermal stability of **Th-PDLLA** with its initial degradation temperature (IDT) value of 243 °C, it can be considered very good if compared to that of commercially available PLA with Mn = 57,000 whose IDT value was of only 215 °C [112]. The same conclusion can be drawn if the temperature of maximum rate of decomposition, (T_dmax_), of 271 °C is taken in consideration, as this characteristic for PLA is usually placed in the range 220 °C–390 °C [113]. Since **Th-PDLLA** is of the oligomeric nature, from these results it can be concluded that by placing of the aromatic thiophene ring as end functionality, the thermal stability of the lactidic oligomer was enhanced [114].

### 3.3. Preliminary Results of Metal-Free Photoinduced Oxidative Homopolymerization of **Th-PDLLA**

Photomediated polymerization takes the advantage of light to allow for a diversified way toward various classes of polymers and macromolecular topologies [115].

Free-radical [116], cationic [117] and anionic chain polymerizations [118] can be performed under irradiation, providing the advantages of technical simplicity, high reaction rates, low energy requirements, milder reaction conditions and temporal and spatial control.

Initiated several decades ago [119], light-mediated synthesis of CPs, by both oxidative and reductive mechanism, give access to p-type and n-type materials [120], being one of the most attractive contemporary approaches [121].

Regarding PTh and its derivatives [75,76,119,122,123], they can be obtained by photoinduced polymerization, employing different reaction conditions. For example, it was previously shown that bare thiophene [75] and 3-hexylthiophene [76] undergo step-growth polymerization by direct photodecomposition of onium salts. The study performed in particular for thiophene [124] revealed that in the presence of diphenyliodonium salt (DPI), the mechanism of polymerization involves successive photoinduced electron transfers (PET) from thiophene to photochemically generated phenyliodinium radical cations, proton release and coupling reactions, finally conducting to PTh. The survey of the literature led us to the conclusion that despite their high potential for practical applications, there are surprisingly few articles dealing with the photochemically induced synthesis of 3-substituted polythiophenes [76,122,123,125]. As such, we decided to attempt the synthesis of a PDLLA-grafted polythiophene by step-growth homopolymerization of **Th-PDLLA** in the presence of DPI via direct PET. The decision was based on the fact that there are practically no differences between the UV–vis absorption of bare thiophene and that of **Th-PDLLA**, and therefore the chosen reaction conditions were adjusted based on those previously reported [75,76].

The changes noticed after 24 h of reaction were obvious; namely, a solid mass with a changed color from the initial white of **Th-PDLLA** (Figure S5A) to a brownish shade and having a glassy appearance was obtained. It is very likely that as the polymerization reaction proceeds the new product formed will not necessarily be insoluble in CH_2_Cl_2_, but due to the increase in amphiphilic character and the change in topology to a branched one the self-assemble into supramolecular structures with rigidification, which eventually leads to “swallowing” of the used solvent, could be possible. The purification of the obtained product was performed by successive washings, extractions and precipitations, as schematically represented in Scheme S1. The insoluble fraction in CH_2_Cl_2_ was retained as the main reaction product denoted as **OTh-PDLLA** (Figure S5B). Being soluble in Chl, the above described behavior of the **OTh-PDLLA** could be explained based on the values of the solvent-polymer interaction parameters (χ) listed in Table S1. An approximately three-fold increase in value of χ can be observed for CH_2_Cl_2_ in comparison with Chl.

The occurrence of photopolymerization process was confirmed by ^1^H-NMR, FTIR and GPC measurements for the main product **OTh-PDLLA**. Moreover, a structural assessment by ^1^H-NMR was performed for the secondary fractions **F1** and **F2** in Scheme S1.

**Figure 4:**
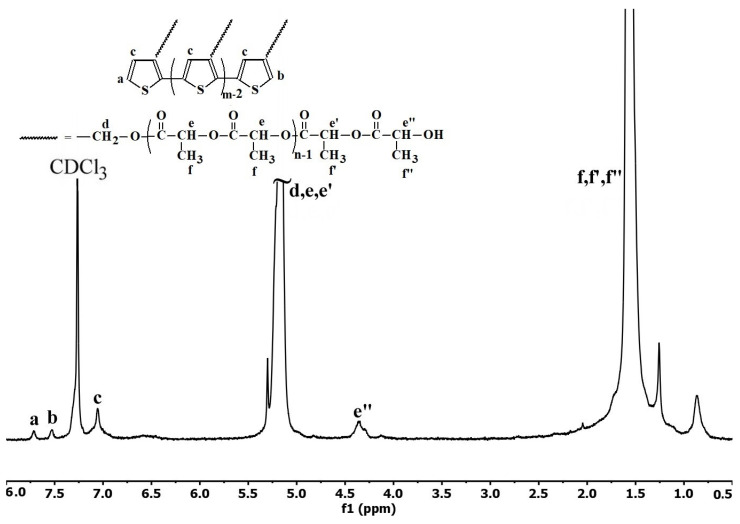
The ^1^H-NMR spectrum of the **OTh-PDLLA** recorded in CDCl_3_.

Thus, one from the evidence for the thiophenic oligomeric conjugated main chain formation is provided by the ^1^H-NMR spectrum in Figure 4. This spectrum features are obviously different when compared with the spectrum of the macromonomer in Figure 1A, in particular in the aromatic region of the spectrum, downfield of 7.5 ppm. Therefore, at 7.71 ppm and 7.53 ppm appeared the characteristic peaks to the protons **a** and **b**, belonging to the thiophene rings at both chain ends (Figure 4), having, as expected, a similar value for the integrated area. As a general feature of the spectrum, the peaks are broader in shape, including that characteristic of protons **c** attributed to the enchained thiophene rings, which appeared at 7.05 ppm upfield shifted when compared with the peak for the proton at the same position of the ring in the macromonomer. Most probably, this small shifting observed for **OTh-PDLLA** is due to the increased electrons density around the enchained thiophene rings, provided by the neighborhood of regularly attached PDLLA side chains rich in electrons due to the ester groups.

Moreover, in the aliphatic region of the spectrum, in the range 5.0–5.5 ppm, the multiplet due to **e**, **e’** and **d** protons has a more complicated shape when compared with that of the macromonomer. The signal for proton **e”** individually appeared centered at approximately 4.36 ppm, being also broader than its homologue in the spectrum of the macromonomer in Figure 1A.

The broadness and shape changes are also kept by the multiplet into the region 1–2 ppm of the spectrum in Figure 4, where the signals of the protons in the aliphatic CH_3_ groups appeared.

What is important to mention is that the ratio of the integral areas of peaks **c** and **e”** has a value close to 1, similar with that obtained for the homologues protons of macromonomer **Th-PDLLA**. This is indirect evidence that PDLLA side chains kept their integrity (chain length) under the condition of applied photoinduced oxidative polymerization.

In addition, by comparing the values of the integrals for the proton **c** with that of the proton **a** or of the proton **b**, a value of 6.9 was obtained; this offers an indication of the PD of the main chain of obtained **OTh-PDLLA**. Such a result was expected, because as already mentioned in the literature, the application of photoinduced oxidative polymerization generally leads to oligomers formation [76,122,125]. Therefore, it can be concluded that the conjugated main chain of **OTh-PDLLA** is not longer than seven structural repeating units, and most probably not shorter than three repeating units. This claim can be supported by the spectra of fractions **F1** and **F2**, (Figure S6), separated during the reaction product purification (Scheme S1). It is easy to observe that the two spectra belong essentially to the compounds that differ from each other, as it was particularly emphasized by the expanded area of the spectrum between 7.4 and 7.8 ppm (see Figure S6). This structural difference is also obviously supported by the presence of the peaks attributable to the protons of the terminal hydroxyl groups of PDLLA side chains, which appeared at different positions in the spectrum of **F1** (2.82 ppm) by comparing with **F2** (3.28 ppm). It can be claimed that the fractions **F1** and **F2** contain the shorter oligomers that were formed during the polymerization reaction, but the presence of the unreacted **Th-PDLLA** macromonomer traces cannot be excluded.

The GPC data in Figure S1C are also indicative for a transformation of **Th-PDLLA** during the applied photoinduced process. A significant increase in M_n_ value of **OTh-PDLLA** was registered in comparison with the macromonomer (from 2463 to 4508). Even if the obtained Mn value suggests rather a dimerization and not the formation of an oligomer, an increase in IPD from 1.2 to almost 2 was also noticed, which is characteristic for a polymer obtained by a step-growth process.

The data obtained by FTIR investigation (Figure S2) of **OTh-PDLLA** complement those of ^1^H-NMR and GPC. As a main structural component of resulting **OTh-PDLLA**, the IR absorption bands associated with PDLLA side chains are all discernible in Figure S2 (black line), at positions which are the same or with a very low shifting (1–2 cm^−1^), with the spectrum keeping its shape. The typical peaks for the thiophene ring are also present in the spectrum. For example, the small but noticeable band at 3112 cm^−1^ is due to aromatic C_α_-H and C_β_-H stretching vibrations, the presence of which supports the claim about the oligomeric nature of the newly formed conjugated main chain [59,123]. The bands at 862, 831 and 699 cm^−1^, listed in Table 1 and assigned to the thiophene ring in the macromonomer, kept their positions and are also present in the spectrum of **OTh-PDLLA**. The newly appeared signals at 892 cm^−1^ and the one centered at approximately 1621 cm^−1^ (both of them marked and enlarged separately in Figure S2) support the transformation and the photoinduced homopolymerization process.

Doubtless the last mentioned band is the most significant, being typical for the conjugation between thiophene rings along the oligomeric chain [59,123].

**Figure 5:**
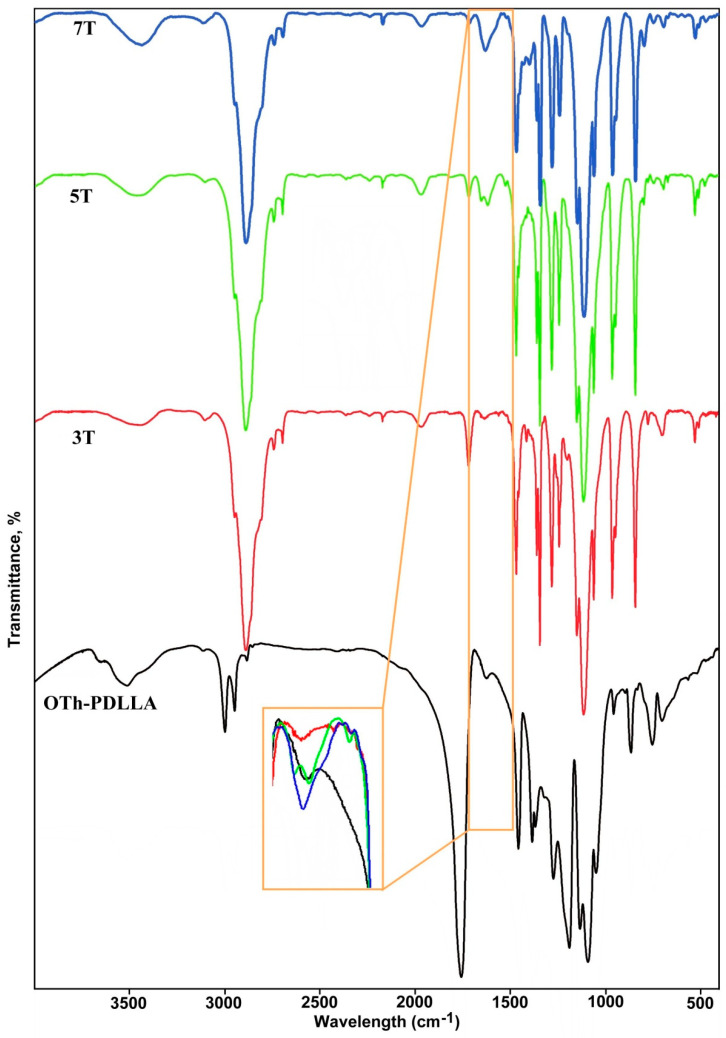
FT–IR spectra of PEG_2000_ -substituted thiophene oligomers containing 3 (**3T**), 5 (**5T**), or 7 (**7T**) thiophene rings and the spectrum of **OTh-PDLLA**.

In order to verify this assertion, in Figure 5 the IR spectrum of **OTh-PDLLA** is presented comparatively with those of three thiophene oligomers reported by us [27,126,127], containing an odd number of thiophene rings (denoted with **3T**, **5T** and **7T** in Figure 5, where the digits represent the number of the thiophene rings), each of them having the central ring substituted in the 3rd position with a PEG 2000 chain.

These oligomers have well-defined length of the conjugated chain, as long as they were obtained by an iterative approach. The range between 1560 and 1680 cm^−1^ of these FTIR spectra was analyzed (the area bounded by a rectangle from Figure 5). It can be seen in the enlarged area in Figure 5 that all the oligomers contain, in the named rage, the absorption bands attributable to the conjugated chains.

Moreover, it can also be noticed that the peak from **OTh-PDLLA** fits very well with that of the pentatiophene **5T**; it might be presumed that the chains having five enchained thiophene rings could be the most abundant component of **OTh-PDLLA**.

**Scheme 2:**
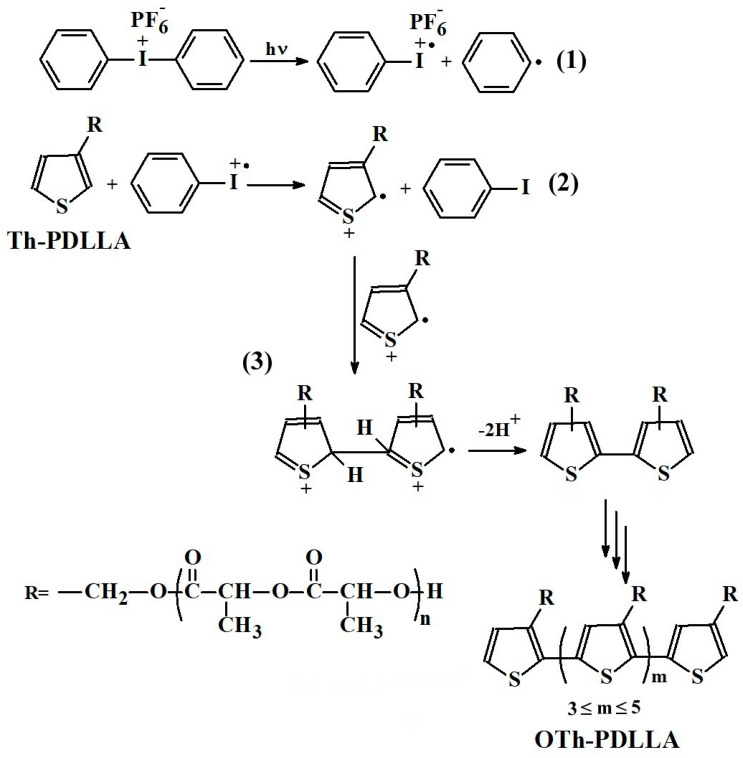
The proposed hypothetical pathway for the synthesis of **OTh-PDLLA** by photo-induced oxidative hopomolymerization of **Th-PDLLA**.

Therefore, based on the structural characterization’s results, and also taking into account the well-known inaccuracies inherent of conventional GPC measurement [61,126], in particular for grafted, branched polymers, which have the more compact structure than the polystyrene used as standard [57], it can be concluded that by photoirradiation in the presence of DPI, **Th-PDLLA** macromonomer underwent photoinduced oxidative homopolymerization with formation of PDLLA-grafted oligothiphene **OTh-PDLLA**. With the above data in hand, and also by considering the previous published articles [75,76,124], in Scheme 2 is advanced a hypothetical pathway for the polymerization of **Th-PDLLA**. Thus, it can be supposed that direct photolysis of DPI (1) results in phenyliodinium radical cations formation, (PhI^+.^), which is subsequently followed by electron transfer reaction from thiophene to form thiophene radical cations (2). By successive proton release and coupling reactions (3), the grafted oligothiophene conjugated main chain is formed in a similar way to that described for electrochemical polymerization [128]. To verify the validity of this hypothesized mechanism and formation of the named reactive species, particular techniques [124] will be necessary to be used in the future.

**Figure 6:**
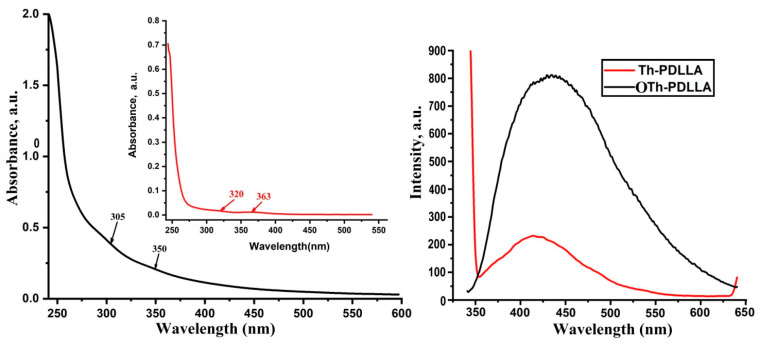
UV–vis and fluorescence spectra of **OTh-PDLLA** in chloroform (c = 1 mg/mL) (λ_ex_ = 330 nm).

Photophysical properties of the newly obtained grafted oligomer **OTh-PDLLA** were investigated as well, and the results are presented in Figure 6.

Several findings of these investigations are worth noting. The first of them is regarding the UV–vis spectrum of the new formed grafted oligothiophene, which shows hardly discernible absorptions at 305 and 350 nm. A similar phenomenon was recently reported by us for another grafted polythiophene [29] and was motivated by the high density of the grafted side chains, which were placed in every structural repeating unit of the polymer, and particularly to those of oligoester nature. For the fluorescence, as expected, the value of the maximum emission wavelength increased from 420 nm in **Th-PDLLA** macromonomer to 436 nm for **OTh-PDLLA** due to the formed conjugated main chain. Remarkable about the fluorescence curve is its large width, which ranges between 350 and approximately 650 nm, but also the fluorescence intensity which increased more than three times.

The value of λ_(em)max_, compared to those reported for thiophene oligomers of different lengths [104], is lower than that of oligomers with chain lengths similar to the one claimed to have been obtained (penta- and/orhexathiophene). This lower value can be motivated, on the one hand, by the electron-withdrawing character of the ester function, from the repeating units of the PDLLA side chains, which is known to produce such an effect [59]. On the other hand, the side chain substitution on neighboring thiophene rings causes oligothiophenes conjugated chain to become non-planar, and consequently to emit at a lower value of wavelength when compared with their bare counterparts. The photophysical properties, especially the fluorescence, of this thiophene oligomer could be also influenced by the possible formation of the supramolecular self-assembled structure mediated by its structural dissymmetrical character and particular architecture, while the PDLLA side chains placed into a confined space of a self-assembled structure could be responsible for, or contribute to, the observed very high increase in the emission intensity around 440 nm.

Regarding the other properties of **OTh-PDLLA**, especially those significant for the biomedical applications for which it was designed (like electrochemical behavior, self-assembling behavior in solvents of various selectivity, surface properties of the derived films, biocompatibility), they are being investigated and will be the subject of a future report.

## 4. Conclusions

The integration of PLA with conjugated CPs is an interesting approach targeting both sustainable electronic devices and various biomedical applications. In this context, the present paper reports on the synthesis, structural characterization and properties in solution and in bulk of a new thiophene-containing electroactive macromonomer derived from oligo-(D,L-lactide).

One of the most important conclusions of the present study is that besides being a building block for the construction of a complex macromolecular architecture, **Th-PDLLA** is a valuable material in itself, demonstrating once more the versatility of the end-functionalization as an alternative way to create materials with new functions from usual polymers. Therefore, designed as an amphiphilic homopolymer, it also behaves as a “shape amphiphile” due to aromatic nature and planar geometry of the thiophene ring contained in its structure. Investigations of **Th-PDLLA** behavior in solvents of different polarity and in bulk shed light on the influence the placing of thiophene ring as α end-group has on the obtained oligo-(D,L-lactide). The results emphasized that this particular amphiphilc property enabled the formation of self-assembled structures in solution, which in turn determined the appearance of non-canonical photoluminescence. In bulk, if the thiophene final group has a minimal influence on the T_g_ value, the thermal stability of lactidic oligomer was definitely enhanced due to its presence. The DSC results suggest that **Th-PDLLA** resulted from synthesis with an enriched isotactic microstructure that could be a consequence of the particular features of **Th-Me** used as initiator.

To the best of our knowledge, for the first time, this study reports on metal-free photoinduced oxidative polymerization of an electroactive macromonomer. The synergistic combination of different techniques for structural investigation demonstrated that polymerization of **Th-PDLLA** occurred by photoirradiation in the presence of DPI as onium salt, with the formation of a conjugated thiophenic main chain of oligomeric nature. It was revealed that its chain length does not exceed seven structural repeating units; very probably, it mostly contains a pentathiophene grafted with PDLLA. In addition, the photophysical properties in the solution of the new synthesized oligothiophene revealed unusual behavior (as a blurred absorption and a high increase in the emission intensity) that could be induced by the “hairy” architecture and by the mismatch between rigid–flexible elements present in its structure.

Future studies are needed to complement the properties of both the macromonomer and the derived grafted oligothiophene, some of them being currently on the way.

## Figures and Tables

**Table 1:** Data from the **Th-PDLLA** FTIR spectrum (Figure S2) ^a^.

Band Position (cm^−1^)	Assignment	Band Position (cm^−1^)	Assignment
3516	υ(OH)	1272	d(C-H) + υ(C-O-C)
***3111***	υ(C-H)_α_+ υ(C-H)b	1188	υ_as_(C-O-C) + r_as_(CH3)
2998	υ_as_ (CH_3_)	1133	r_as_(CH3)
2943	υ_s_ (CH_3_)	1090	υ_s_(C-O-C)
2880	υ(CH)	1046	υ_s_(C-CH_3_)
1755	υ (C=O)	955	r(CH3) + υ(C-C)
***1617***	υ(C=C)_ring_	916	r(CH3) + υ(C-C)
1457	d_as_(CH_3_)	***862***	υ_as_(C-S)_ring_
1383	d_s_(CH_3_)	***831***	υ_s_(C-S)_ring_
1366	d_1_(C-H) + d_s_(CH_3_)	***795***	g(C-H)
1321	d_2_(C-H)	***699***	d(C-S)

^a^—in **Bold** and ***Italics*** are the typical values for the thiophene ring.

## Data Availability

The data presented in this study are available on request from the corresponding authors.

## Supplementary Materials

The following supporting information can be downloaded at: https://www.mdpi.com/article/10.3390/polym15051094/s1. Figure S1. The GPC traces for **Th-PDLLA** macromonomer in THF (A) or chloroform (B) and GPC trace of **OTh-PDLLA** in chlorfom; Figure S2. The IR spectra of **Th-PDLLA** macromonomer (red line) and of PDLLA-substituted oligothiophene, (**OTh-PDLLA**), obtained by photo-induced oxidative polymerization of **Th-PDLLA** (black line); Figure S3. DLS trace of intensity-weighted distribution of apparent hydrodynamic diameter (Dh) for **Th-PDLLA** in Chl at concentration of 1 mg/mL; Figure S4. DSC traces of **Th-PDLLA** macromonomer; Figure S5. Photographs of (A) **Th-PDLLA** macromonomer freshly dried powder and of the resulted homopolymer **OTh-PDLLA** (B); Figure S6. ^1^H-NMR spectra in CDCl_3_ of the fractions resulting during the purification of the **Th-PDLLA**’s photopolymerization reaction product: **F1** (black line) and **F2** (red line) (see Scheme S1 for details identification); Scheme S1. Purification and separation steps of the reaction product obtained by **Th-PDLLA**’s photopolymerization; Table S1. Several physical properties of the used solvents for investigations and those of the **Th-PDLLA** macromonomer’s constitutive parts; Supplementary comment regarding the possible behavior of the macromonomer in THF; more details can refer to Refs. [129,130,131,132,133,134,135,136,137,138,139,140,141]; Supplementary References.
