# Supplementary material for: Thiophene End-Functionalized Oligo-(D,L-Lactide) as a New Electroactive Macromonomer for the “Hairy-Rod” Type Conjugated Polymers Synthesis
Source: Polymers (Basel). 2023 Feb 22;15(5):1094. doi: 10.3390/polym15051094 (PMC10006927; doi:10.3390/polym15051094)
Supplement: Supplementary file 1 [file polymers-15-01094-s001.zip › polymers-2221669-supplementary.pdf]

Thiophene End-Functionalized Oligo-(D,L)-Lactide as a New Electroactive Macromonomer for the "Hairy-Rod" Type Conjugated Polymers Synthesis

Anca-Dana Bendrea [1], Luminita Cianga [1,*], Demet Göen Colak [2], Doina Constantinescu [3] and Ioan Cianga [1,*]

[1] "PetruPoni" Institute of Macromolecular Chemistry, Centre of Advanced Research in Bionanoconjugates and Biopolymers, 41A, Grigore–GhicaVoda Alley, 700487 Iasi, Romania
[2] Department of Chemistry, Faculty of Science and Letters, Istanbul Technical University, 34469 Istanbul, Turkey
[3] MONOFIL SRL, Str. Gheorghe Caranfil nr. 5F, 617410 Savinesti, Romania
* Correspondence: lcianga@icmpp.ro (L.C.); ioanc@icmpp.ro (I.C.); Tel.: +40-332-880-220 (L.C. & I.C.)

Supporting Information

CONTENT

Supplementary Figures and Scheme.. 2

Supplementary Table.. 5

Supplementary comment regarding the possible behavior of the macromonomer **Th-PDLLA** in THF.......6

Supplementary References... 6

Supplementary Figures and Schemes

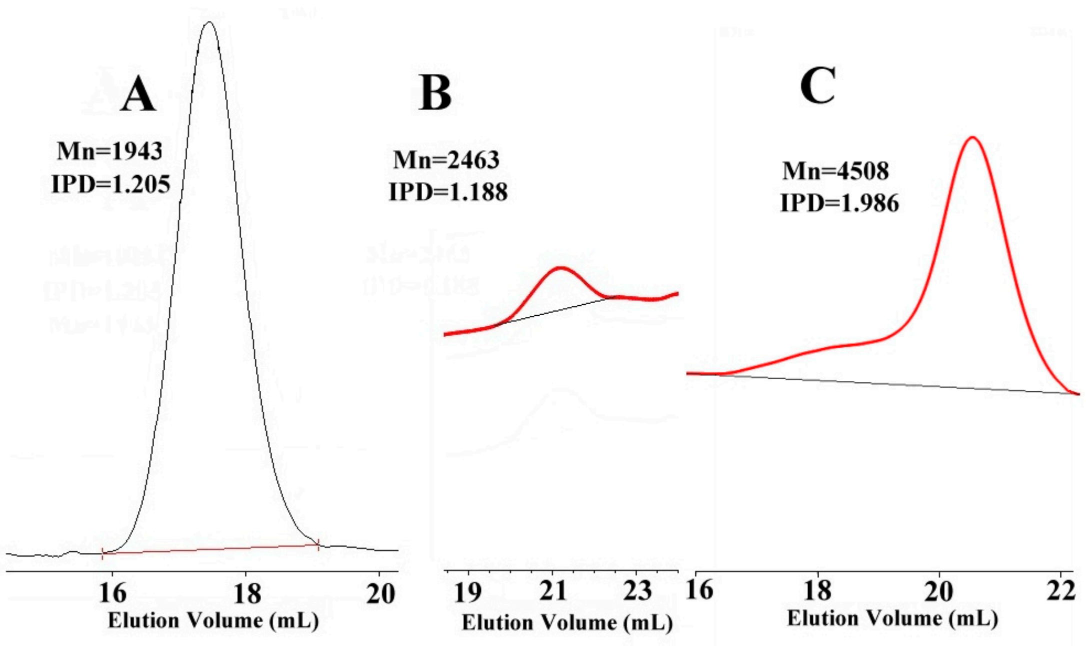

Figure S1. The GPC traces for **Th-PDLLA** macromonomer in THF (**A**) or chloroform (**B**) and GPC trace of **OTh-PDLLA** in chlorfom

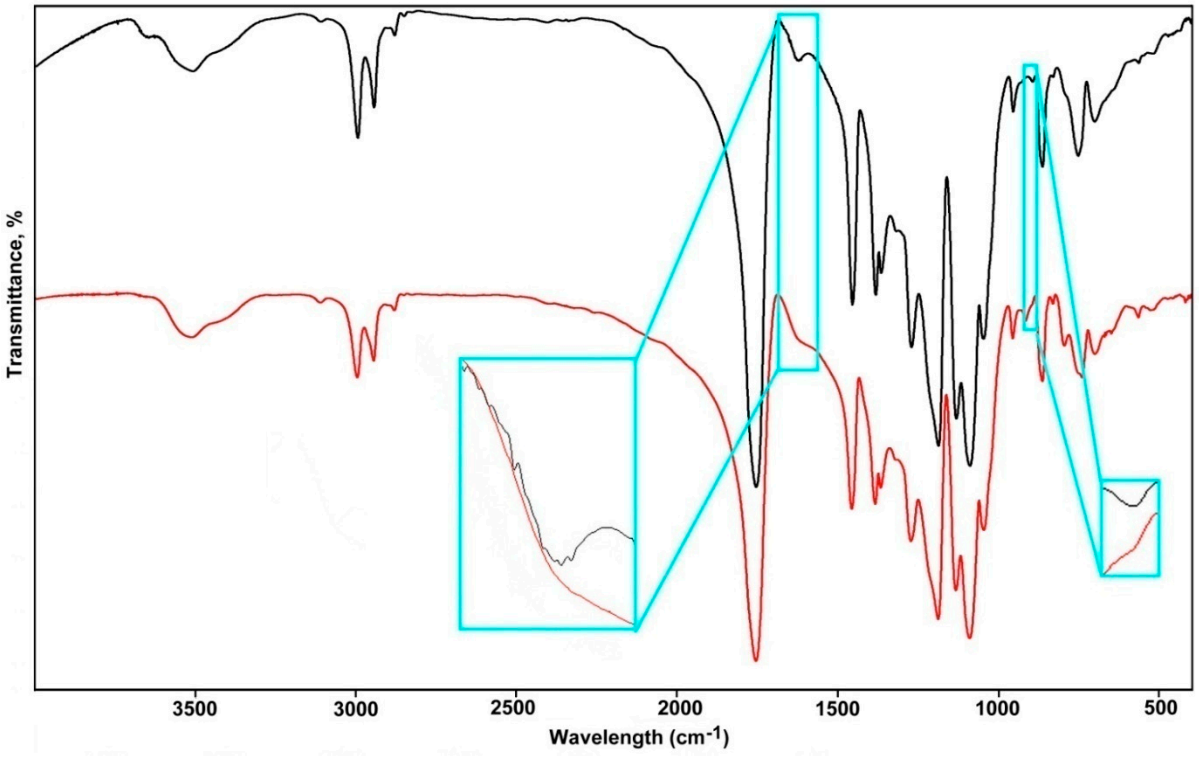

Figure S2. The IR spectra of **Th-PDLLA** macromonomer (red line) and of PDLLA-substituted oligothiophene, (**OTh-PDLLA)**, obtained by photo-induced oxidative polymerization of **Th-PDLLA** (black line)

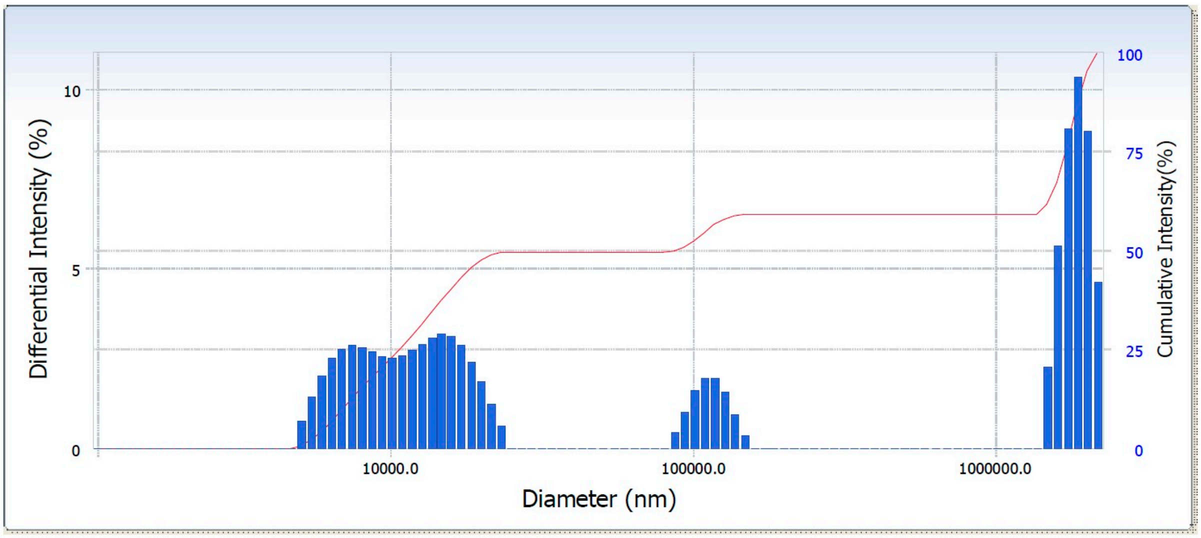

Figure S3. DLS trace of intensity-weighted distribution of apparent hydrodynamic diameter (D_h) for **Th-PDLLA** in Chl at concentration of 1mg/mL

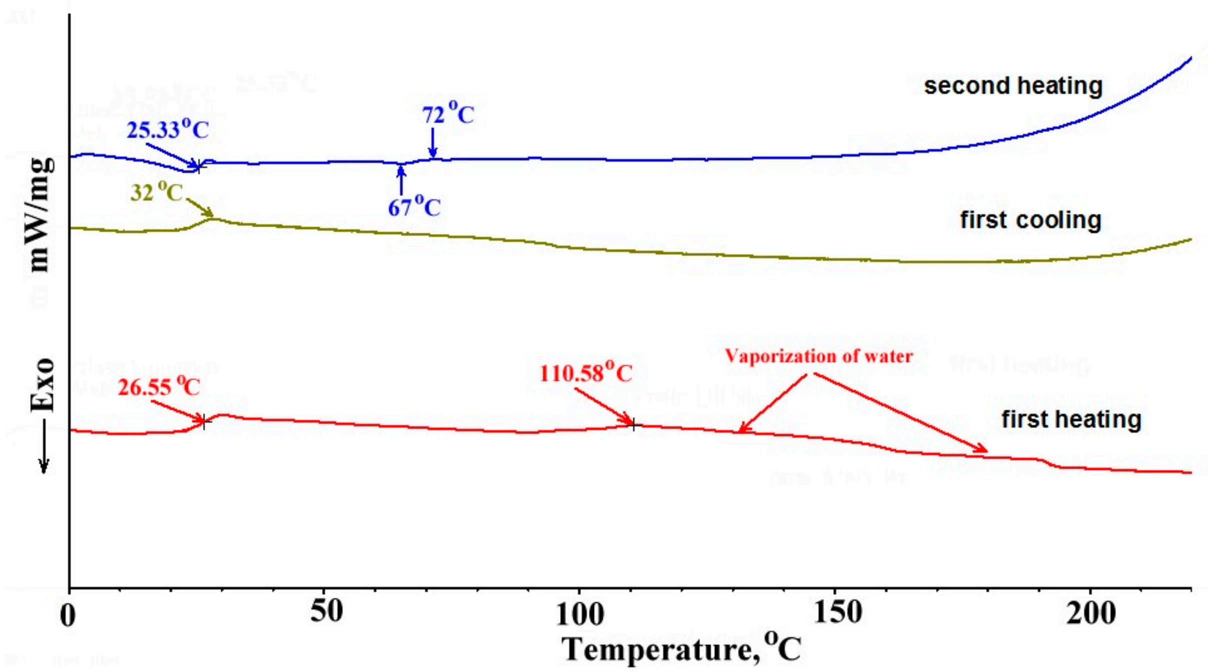

Figure S4. DSC traces of **Th-PDLLA** macromonomer

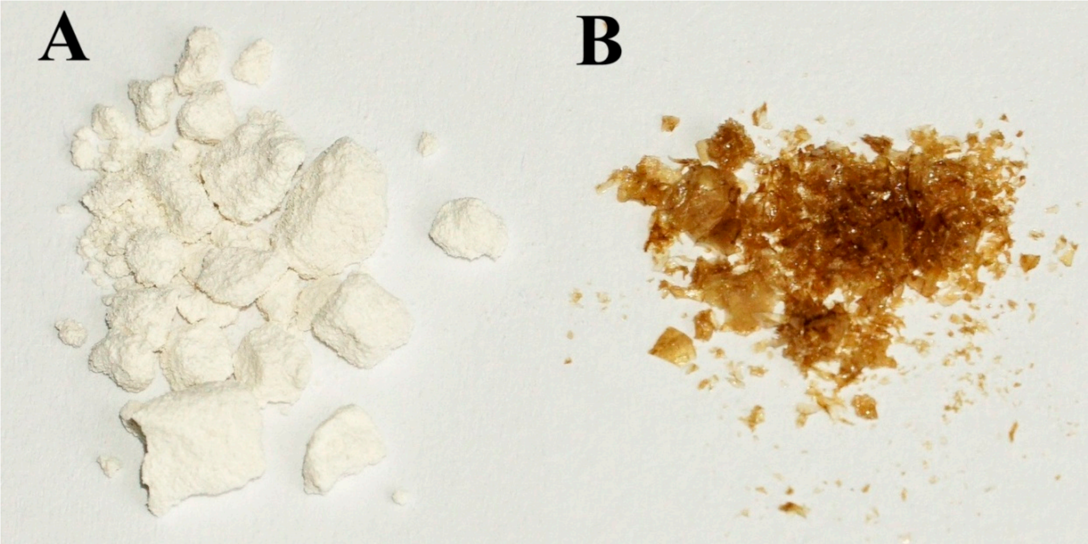

Figure S5. Photographs of (**A**)- **Th-PDLLA** macromonomer freshly dried powder and of the resulted homopolymer **OTh-PDLLA** (**B**)

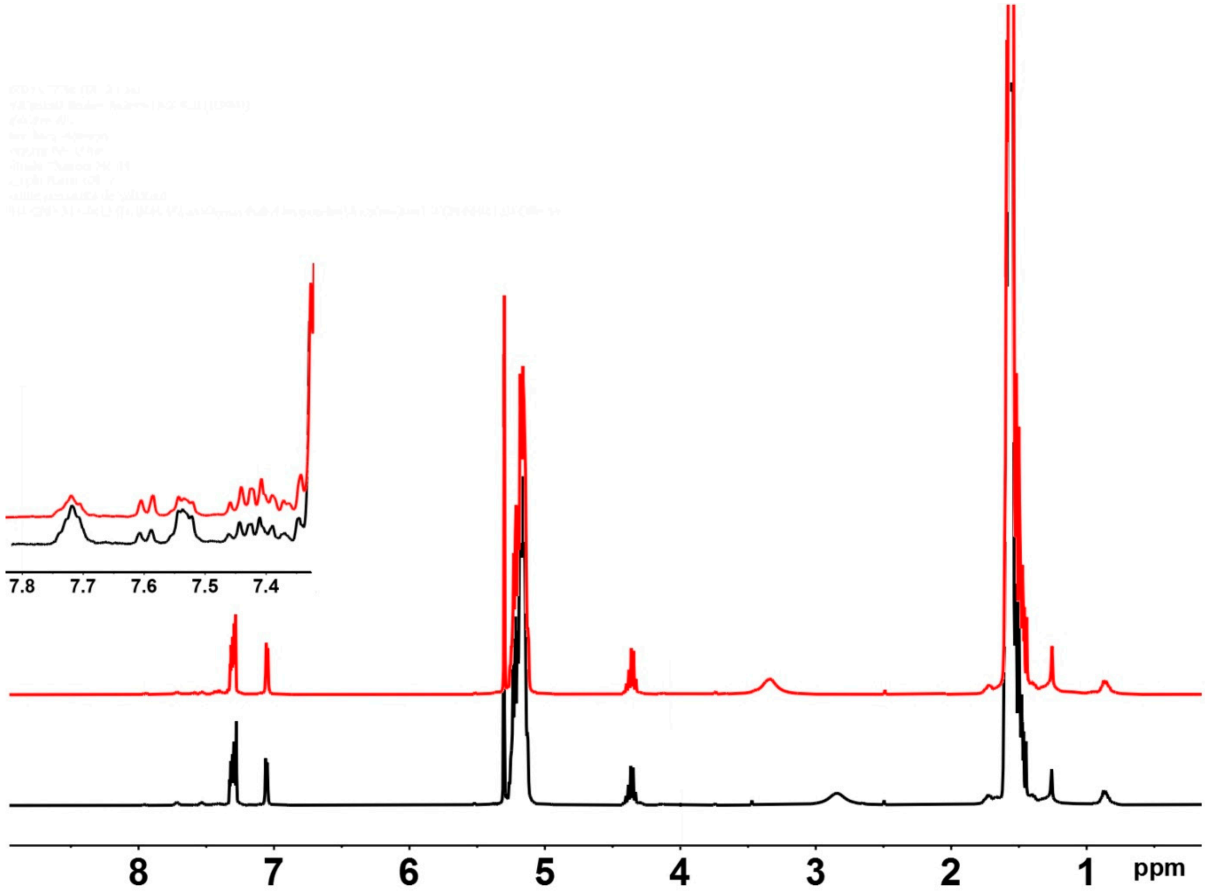

Figure S6. ^{1}H-NMR spectra in $CDCl_3$ of the fractions resulting during the purification of the **Th-PDLLA**'s photopolymerization reaction product: **F1** (black line) and **F2** (red line) (see Scheme S1 for details identification)

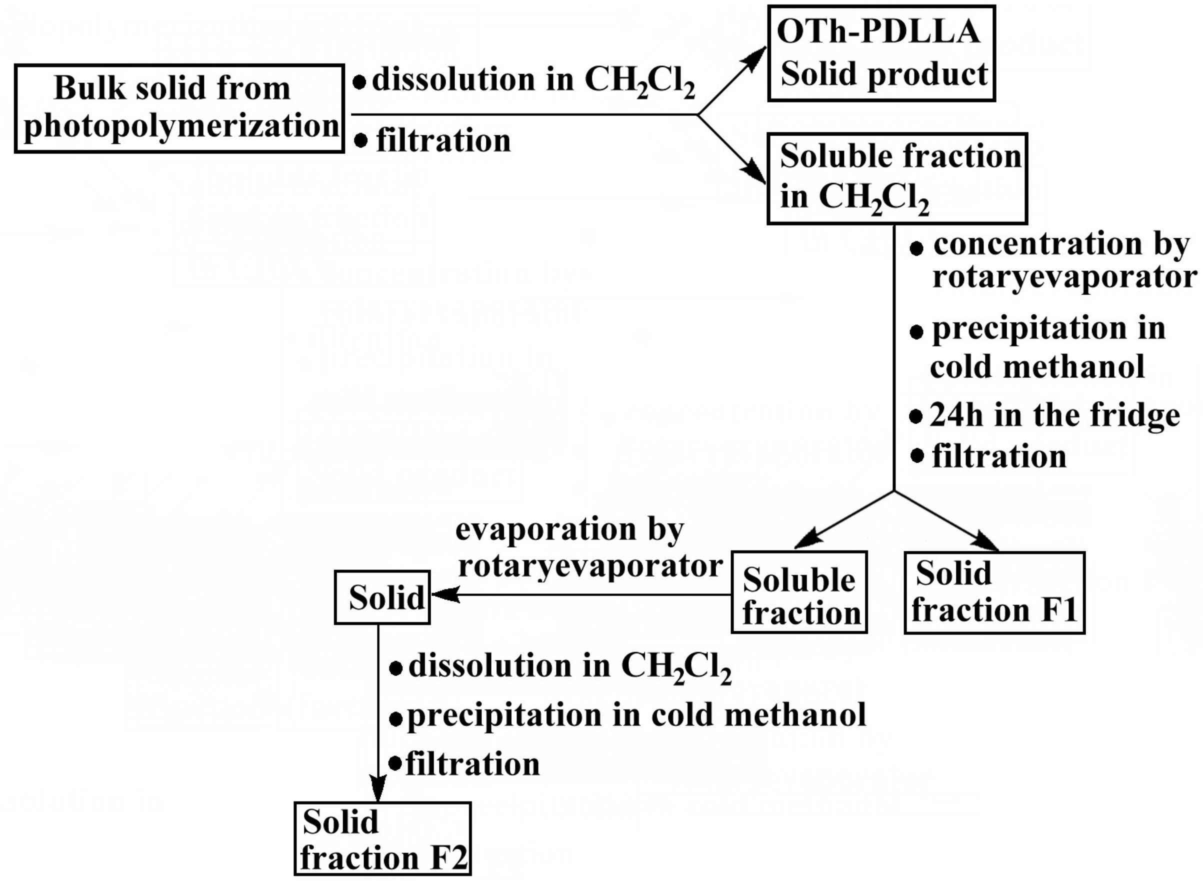

Scheme S1. Purification and separation steps of the reaction product obtained by Th-PDLLA's photopolymerization

Supplementary Table

Table S1. Several physical properties of the used solvents for investigations and those of the **Th-PDLLA** macromonomer's constitutive parts[*]

Compound (e)[&]	Hansen Solubility parameters (MPa ½)				Solvent-Polymer interaction parameters (χ)			
	δ_d	δ_p	δ_H	δt	Chl	THF	ACN	CH_2Cl_2
**PDLLA**	18.6[1]	9.9[1]	6[1]	22[2]/20.5[3]	0.32[6]	-	0.43[6]	0.99[6]
**Thiophene;(2.86)**	18.9[3]	2.4[3]	7.8[3]	20[4]	miscible[#]	miscible[#]	miscible[#]	
**Chl;(4.81)**	17.8[5]	3.1[5]	5.7[5]	19[5]	-	-	-	
**THF;(7.52)**	16.8[5]	5.7[5]	8[5]	19.4[5]	-	-	-	
**ACN;(36.6)**	15.3[5]	18[5]	6.1[5]	24.3[5]	-	-	-	
**CH_2Cl_2;(8.35)**	18.2[5]	6.3[5]	6.1[5]	19.8[5]				

*- the superscript numbers in the table correspond to the similar numbers in the References section; #- experimentally noticed; [&]- dielectric constant

Supplementary comment regarding the possible behavior of the macromonomer Th-PDLLA in THF

Reported as being partially soluble in THF [8], however PDLLA samples of different molecular weight were evaluated by GPC using THF as eluent [9,10].

What it is really relevant, it is that a recent study [11] demonstrated that of PDLLA in THF behaves as a typical flexible polymer but it is essentially 1.6−2.1 times stiffer than the representative vinyl polymers, (polystyrene or poly(methyl methacrylate)), most likely due to the planar feature of the ester linkage, which partially constrains the free rotation of the main chain. If the traces of the **Th-PDLLA** absorbance in Figure 2A (main manuscript) are considered, the noticed differences, in particular when comparing that in THF with the one obtained in Chl, could be expectable, as the oligolactide chains changed their flexibility, being stiffer in the former solvent.

Moreover, our experimental findings intuitively suggest the formation of self-assembled supramolecular structures also in THF. The presence of eventually dispersed particles having the size comparable with the incident wavelenght could sits at the base of the light scattering phenomenon [12]; which could be supported by the high increase of the apparent absorbance intensity around 300 nm (Figure 2A) [12].

However, to clarify the different and particular behaviour of **Th-PDLLA** in THF and the less usual shape of the UV-vis trace in this solvent, a more deep and particularly devoted study in the futureis needed, all the more so as the photo-physical properties of PDLLA in the presence of additives seems not to be a trivial issue [13].

References

1. S. Abbott, Chemical compatibility of poly(lactic acid): A practical framework using Hansen solubility parameters, in Poly(lactic acid) Synthesis, Structure, Properties, Processing, and Application, R. Auras, et al., Editors. 2010, John Wiley and Sons, Inc.: Hoboken, New Jersey. p. 83-89.

2. K. Adamska, A. Voelkel, A. Berlinska, The solubility parameter for biomedical polymers-Application of inverse gas chromatography, *J. Pharm. Biomed*., 2016, 127, 202-206.

3. U. Siemann, The solubility parameter of poly(DL-lactic acid), Eur. Polym. J., 28, 293-297, 1992.

4. Charles M. Hansen a, Allan L. Smith, Using Hansen solubility parameters to correlate solubility of C60 fullerene in organic solvents and in polymers, *Carbon* 42 (2004) 1591–1597.
5. Kun Liu, Chuan-Jie Fang, Zhi-Qiang Li, Matthew Young, Separation of thiophene/n-heptane mixtures using PEBAX/PVDF-composited membranes via pervaporation, *J. Membr. Sci*. 451 (2014) 24–31.
6. A. F. Barton, Solubility Parameters, *Chem. Rev*., 1975, 75, 731-753.
7. Orwoll, R.A., Arnold, P.A. (2007). Polymer–Solvent Interaction Parameter χ. In: Mark, J.E. (eds) *Physical Properties of Polymers Handbook*. Springer, New York, NY. https://doi.org/10.1007/978-0-387-69002-5_14.
8. Casalini T, Rossi F, Castrovinci A and Perale G A Perspective on Polylactic Acid-Based Polymers Use for Nanoparticles Synthesis and Applications, *Front. Bioeng. Biotechnol.* 2019, 7, 259. doi: 10.3389/fbioe.2019.00259

9. van Dijk JAPP, Smit JAM, Kohn FE, Feijen J. Characterization of poly(D,L-lactic acid) by gel permeation chromatography. *J Polym Sci Polym Chem Ed*. 1983, 21,197–208.

10. Othman N, Acosta-Ramírez A, Mehrkhodavandi P, Dorgan JR, Hatzikiriakos SG. Solution and melt viscoelastic properties of controlled microstructure poly(lactide*). J Rheol*. 2011, 55, 987–1004.

11. Suzuki, Y., Watanabe, T., Kosugi, H., Ueda, K., Kikuchi, M., Narumi, A., Kawaguchi, S., Dilute solution properties of poly(D,L-lactide) by static light scattering, SAXS, and intrinsic viscosity *Polym. J*., 2020, 52, 387–396.

12. Noble, J. E., Quantification of Protein Concentration Using UV Absorbance and Coomassie Dyes in *Methods in Enzymology*, vol. 536, chapter 2, 17-26, Elsevier Inc. 2014, http://dx.doi.org/10.1016/B978-0-12-420070-8.00002-7., pp. 22-23.

13. Zhang, S., Liu, T., Zhao, B., Verdi, C., Liu, W., Hao, C., Zhang, J., Shape memory Poly(lactic acid) binary blends with unusual fluorescence, *Polymer*, 2020, 209, art no. 122980.
